# Visualizing changes of metabolites during iron deficiency chlorosis in field-grown pear leaves using micro-Raman spectral imaging

**DOI:** 10.3389/fpls.2022.1079660

**Published:** 2023-01-12

**Authors:** Zhen Gao, Chunjiang Zhao, Daming Dong, Songzhong Liu, Xuelin Wen, Yifan Gu, Leizi Jiao

**Affiliations:** ^1^ College of Information and Electrical Engineering, China Agricultural University, Beijing, China; ^2^ National Research Center of Intelligent Equipment for Agriculture, Beijing Academy of Agriculture and Forestry Sciences, Beijing, China; ^3^ Institute of Forestry & Pomology, Beijing Academy of Agriculture & Forestry Sciences, Beijing, China; ^4^ Wuhan National Laboratory for Optoelectronics (WNLO), Huazhong University of Science and Technology, Wuhan, Hubei, China

**Keywords:** iron deficiency, pear tree, Raman spectroscopy, spectral imaging, chlorosis

## Abstract

Owing to iron chlorosis, pear trees are some of the most severely impacted by iron deficiency, and they suffer significant losses every year. While it is possible to determine the iron content of leaves using laboratory-standard analytical techniques, the sampling and analysis process is time-consuming and labor-intensive, and it does not quickly and accurately identify the physiological state of iron-deficient leaves. Therefore, it is crucial to find a precise and quick visualization approach for metabolites linked to leaf iron to comprehend the mechanism of iron deficiency and create management strategies for pear-tree planting. In this paper, we propose a micro-Raman spectral imaging method for non-destructive, rapid, and precise visual characterization of iron-deficiency-related metabolites in pear leaves. According to our findings, iron deficiency significantly decreased the Raman peak intensities of chlorophylls and lipids in leaves. The spatial distributions of chlorophylls and lipids in the leaves changed significantly as the symptoms of iron insufficiency worsened. The technique offers a new, prospective tool for rapid recognition of iron deficiency in pear trees because it is capable of visual detection of plant physiological metabolites induced by iron deficiency.

## 1 Introduction

By the middle of this century, there will be significant food shortages as the world’s population expands. To meet rising demands for food, agricultural yields must be increased (Pretty et al., 2010). Meanwhile, 30% of the global population suffers from iron-deficiency anemia, which is induced by inadequate iron consumption and low iron bioavailability (Kassebaum et al., 2014). Iron is a crucial trace metal for plants and is necessary for both photosynthesis and chlorophylls synthesis. A substantial decline in fruit productivity and quality will be caused by iron deficiency because it will cause chlorosis, lower photosynthesis and respiration rates, and inefficient water use (Larbi et al., 2006). Pear trees are some of the most severely affected by iron deficiency, and large losses occur each year because of chlorosis (Sanz et al., 1993; Therby-Vale et al., 2022). Therefore, timely detection of iron deficiency in pear trees is crucial for improving the healthy growth of pear trees, fruit quality, and planting efficiency.

The standard approaches for the detection of iron content in leaves, atomic absorption spectroscopy and inductively coupled plasma-emission spectrometry, can accurately measure the total iron content in leaves, but these operations are complicated, time-consuming, and labor-intensive (Kucukbay and Kuyumcu, 2014; Elango et al., 2021). Furthermore, there are limitations to using the total iron content of leaves to discriminate the iron deficiency status of plants. Studies have shown that iron-deficient leaves with intervein chlorosis have total iron contents similar to those of iron-sufficient leaves, which is known as the “chlorosis paradox” (Morales et al., 1998; Romheld, 1998; Jimenez et al., 2009). Therefore, using the total iron content to determine whether leaves are iron deficient is inaccurate.

A chlorotic effect is caused by changes in metabolites, such as pigments in the veins and leaf mesophyll, which are driven by iron deficiency. As a result, occurrences of leaf iron deficiency can be quickly determined using the quantity of chlorophylls and other leaf metabolites (Li et al., 2006). Therefore, some researchers have investigated the use of spectral reflectance to assess the concentrations of leaf metabolites in plants. They discovered that plant chlorosis is more strongly related to active iron content than total iron content (Basayigit et al., 2015). Although reflectance spectroscopy may identify iron deficiency in plant leaves quickly, the wavelength band used is in the visible–near-infrared range. Molecular compounds like chlorophylls have no fingerprints in this spectral range. Therefore, this technology cannot specifically identify molecules such as chlorophylls; instead, it relies on stoichiometric algorithms for modeling and identification, which are low-migration and imprecise.

As a next-generation detection technology for agricultural applications, Raman spectroscopy is advantageous because it allows specific, multi-component analysis, is non-destructive, and rapidly detects molecular compounds (Lew et al., 2020). Obvious physiological changes caused by iron deficiency are significant decreases in the content of leaf metabolites, such as chlorophylls and lipids (Morales et al., 1991). The water-insensitive nature of Raman spectroscopy enables the detection of these metabolites without pre-processing in plant leaves. More importantly, Raman spectroscopy can be integrated with a microscope to form a micro-Raman spectrometer, which can generate maps of relative content distributions of relevant leaf metabolites at a microscopic scale (Baranski et al., 2005; Gierlinger et al., 2008; Heiner et al., 2018; Zhang et al., 2020; Sasani et al., 2021).

In this paper, we conducted micro-Raman spectroscopy on iron-deficient pear leaves. The variation of metabolites, including chlorophylls and Lipids, in leaves affected by iron-deficiency was studied, providing a novel approach for revealing patterns of spatio-temporal variation and mechanisms of changes in metabolites accompanying iron deficiency.

## 2 Materials and methods

### 2.1 Materials and instruments

In North China, we discovered that a high-quality pear cultivar (*Pynus bretschneideri* Rehd.) grafted to quince A (Hardy as interstock) suffered from iron deficiency chlorosis in calcareous soil in early spring, and that the condition was even worse in late spring to early summer. As a result, a high-density training system has been developed in Beijing research and demonstration pear orchard since 2016. This orchard is located in Beijing, China, 40 meters above sea level in the continental monsoon climate zone. The annual average temperature is 10°C, and rainfall occurs mainly from July to September, with an annual average precipitation of 550 mm. The soil is a silt loam consisting of clay, silt, and sand in proportions of 5.4%, 64.7%, and 29.9%, respectively (Zhao et al., 2020).

When iron fertilizer is sprayed on the leaves of yellow pear trees, the leaves can partially return to green, indicating that the yellow symptoms are caused by iron deficiency. To test the feasibility of Raman spectroscopy for visual characterization of iron-deficient leaf metabolites, basal leaves, young leaves, and apical leaves were picked from the same branch of Huangguan (*Pyrus bretschneideri* Rehd) pears with iron-deficiency symptoms, representing healthy, mildly iron-deficient, and severely iron-deficient leaves, respectively (Rustioni et al., 2018). This is because the degree of leaf iron deficiency varies at different positions on a branch, with iron deficiency first occurring on the youngest leaves at the top (Bertamini et al., 2002; Tremblay et al., 2012). The experiment included three groups of biological repetitions, totaling nine leaf samples.

Chlorophylls content within plant leaves is often characterized by soil–plant analysis development (SPAD) values. We used a handheld SPAD meter (SPAD-502, Konica Minolta Sensing, Inc., Osaka, Japan) to measure the SPAD values of healthy, mildly iron-deficient, and severely iron-deficient leaves to provide a reference for the analysis of the Raman spectral results. SPAD was measured three times per leaf.

The micro-Raman spectrometer (HORIBA HR Evolution, Horiba, Japan) can acquire high-resolution Raman spectra from leaves, because of its 800-mm focal length. During the experiment, a 532-nm continuous laser (100-mW power) was the excitation light source, the grating was set to 600 l/mm, the ND filter was set to 3.2%, the single-point integration time was set to 0.5 s, and the single-point accumulation number was set to 1. For the mapping, an area of 500μm by 500μm and 25μm steps were chosen, and every pixel corresponds to one scan. These settings ensure non-destructive Raman spectroscopic measurements of plant leaves.

### 2.2 Data acquisition and analysis methods

**Figure 1:**
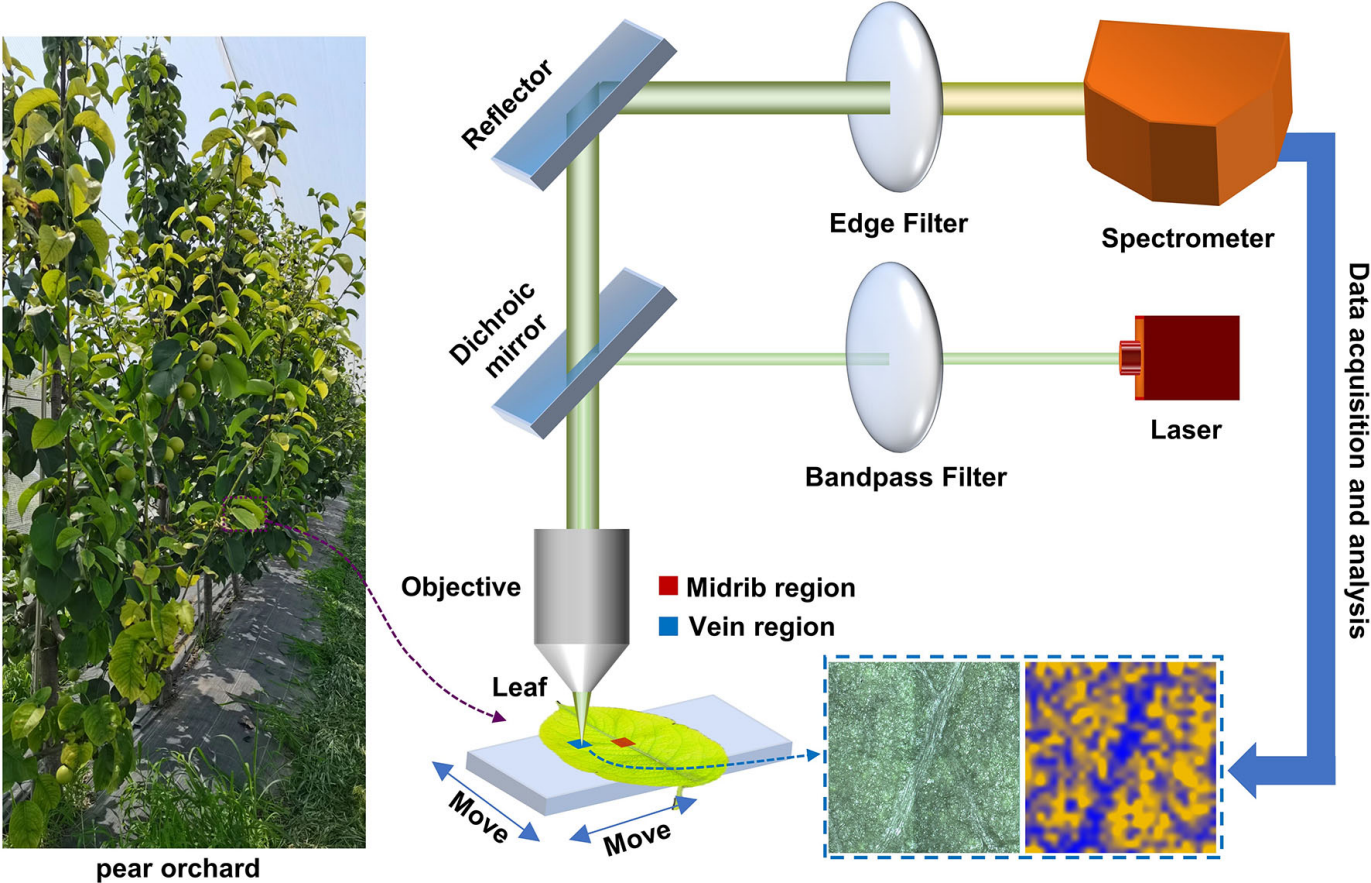
Schematic diagram of spectral-imaging measurement principles of the micro-Raman spectrometer used for pear leaves.

The Raman spectroscopic measurement process for pear leaves is shown in **Figure 1**. Leaves picked from the pear orchard were placed in a portable refrigerator and sent to the laboratory for micro-Raman spectroscopic analysis within 1 hour. Two regions, the midrib and vein, were selected for Raman spectral imaging in each of the healthy, mildly iron-deficient, and severely iron-deficient leaves. Through the displacement of the x-axis and y-axis of the object platform, the Raman hyperspectral data were acquired for the leaf regions. Using a simple characteristic band-spectral imaging method, pseudo-color maps of the content distributions of specific substances in leaves can be obtained quickly. All spectral data were processed using Python. The resulting graph was drawn with origin software and PowerPoint (PPT).

To analyze the Raman spectra, cosmic rays were removed firstly. Because the leaf is a complex matrix, it contains many fluorescent substances. Therefore, under the excitation by visible light (532 nm), interference by fluorescence signals caused a baseline shift of the Raman spectrum of the leaves. For data analysis, we used adaptively iteratively reweighted penalized least squares (airPLS) for baseline correction (Zhang et al., 2010). The corrected Raman spectra were then filtered using the Savitzky-Golay filter method with an order of 1 and a number of points of 3. The relative concentration analysis was based on baseline-corrected, smoothed spectra. All spectrum was maximum normalized. In order to evaluate the variability in intensity of the Raman features of the spectra of the leaves (healthy, mildly iron-deficient and severely iron-deficient), pseudo-color maps based on the intensity of the Raman band was generated using Python. Statistical analysis was carried out with Excel. ANOVA was used to compare individual peaks between iron-deficient and healthy leaves.

## 3 Results

### 3.1 Raman spectral characteristics of metabolites in leaves with different degrees of iron deficiency

**Figure 2:**
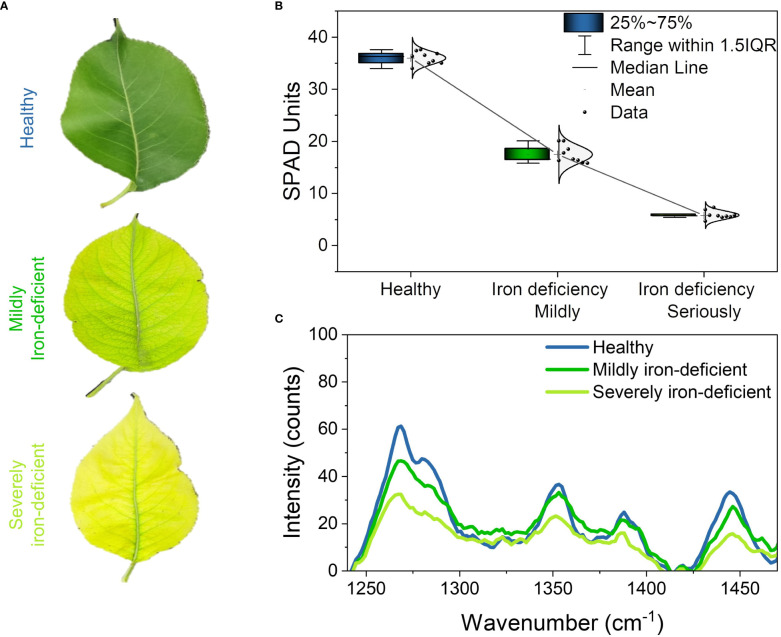
Phenotypes, SPAD values, and Raman spectra of pear leaves with different levels of iron deficiency: **(A)** phenotypes of healthy, mildly iron-deficient, and severely iron-deficient leaves; **(B)** SPAD values of leaves; **(C)** Raman spectra of leaves.

**Figure 2A** depicts the phenotype of the tested leaves. The healthy leaves are dark green in color and have white veins. Iron-deficient leaves are yellow–green in color overall and greenish near the veins. The chlorophylls pigment in leaves gradually decreased as iron deficiency worsened, and the leaves gradually changed from yellowish green to yellow, exhibiting severe symptoms of iron deficiency and chlorosis. Changes in leaf chlorophylls contents caused by iron deficiency were also confirmed by SPAD measurements (**Figure 2B**). The figure shows that the SPAD values of healthy, mildly iron-deficient, and severely iron-deficient leaves were approximately 35, 17, and 5, respectively. The results showed that as iron deficiency worsened, the SPAD value of leaves decreased gradually, as did leaf chlorophylls contents (Yamamoto et al., 2002).

**Figure 2C** depicts the characteristic peaks analysis of the leaf Raman spectra. We found four spectral peaks in the average Raman spectrum of leaves: 1286cm^−1^,1353cm^−1^, 1266cm^−1^, and 1444cm^−1^. **Table 1** shows the attribution of peaks. The Raman peaks at 1286 cm^−1^ and 1353 cm^−1^ were assigned to chlorophylls (Cai et al., 2002; Mandrile et al., 2019), while those at 1266 cm^−1^ and 1444 cm^−1^ were assigned to lipids (Czamara et al., 2015). The intensities of the Raman peaks at 1286 cm^−1^ and 1353 cm^−1^ decreased sequentially in healthy, mildly iron-deficient, and severely iron-deficient leaves, as shown in **Figure 2C**, indicating that relative leaf chlorophylls content gradually decreased as the degree of iron-deficiency worsened. This result is consistent with the SPAD values shown in **Figure 2B**. Furthermore, the intensities of the Raman peaks at 1266 cm^−1^ and 1444 cm^−1^ gradually decreased with increasing severity of iron deficiency. This suggests that iron deficiency causes decreases in both chlorophylls and lipids. This is because iron deficiency reduces the soluble lipids content of the epidermis and also lipids in the vesicle membranes. The variations in characteristic chlorophylls and lipids peaks detected by Raman spectroscopy were correlated with the degree of iron deficiency in leaves according to cross-analyses with leaf phenotypes and SPAD values. Finally, Raman spectroscopy could detect physiological changes caused by iron deficiency in pear trees in a non-destructive and timely manner.

**Table 1:** Vibrational Bands and Their Assignments for Pear Leaf Samples.

band	vibrational assignment
1266	δ(=CH) (lipids)^(^ Czamara et al., 2015 ^)^
1286	δ(phenyl−OH) (phenolics)^(^ Gill et al., 1970 ^)^ + −δ(CH)·ν(CN) (chlorophylls)^(^ Cai et al., 2002 ^)^
1353	undefined (chlorophylls)^(^ Cai et al., 2002 ^;^ Mandrile et al., 2019 ^)^
1444	α(CH_2_/CH_3_) (lipids) ^(^ Czamara et al., 2015 ^)^

### 3.2 Spatial distribution of metabolites around the leaf midrib at different levels of iron deficiency

**Figure 3:**
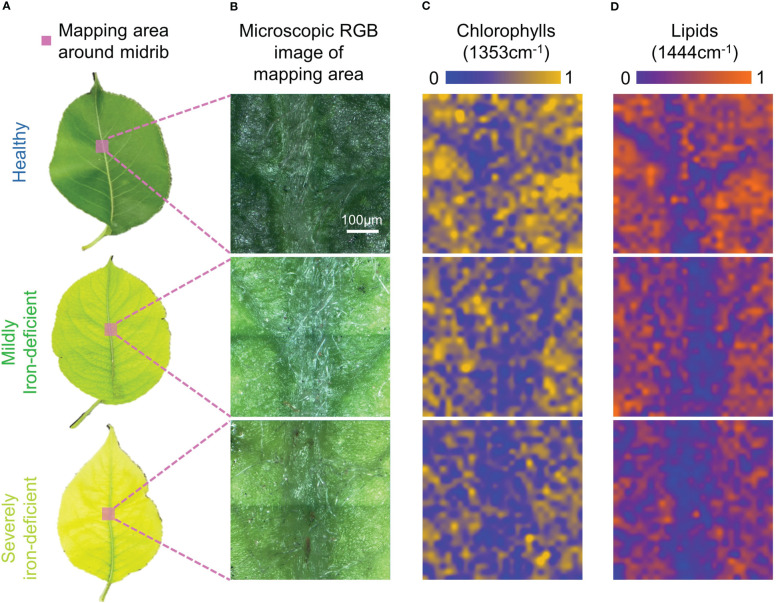
Visible light images and Raman spectral images of midrib components in leaves with differing degrees of iron deficiency. **(A)**Visible light images of healthy and iron-deficient leaves; **(B)** Microscopic visible light images of the midrib-mapping regions; **(C)** Pseudo-color maps of chlorophylls spatial distribution in the midrib-mapping regions; and **(D)** Pseudo-color maps of lipids spatial distribution in the midrib-mapping regions.

**Figure 3** depicts the Raman spectral imaging results of metabolites in the regions near the midribs of leaves with varying degrees of iron deficiency. In mildly and severely iron-deficient leaves, the intervein was chlorotic, whereas the area near the midrib and the veins remained green, as seen in the microscopic images. This is consistent with symptoms of interveinal chlorosis associated with iron deficiency (Bertamini et al., 2002).

Based on the characteristic Raman peak of chlorophylls at 1353cm^−1^, pseudo-color maps of relative chlorophylls content distributions near the midribs were generated. In **Figure 3C**, the chlorophylls content in the mesophyll region of healthy leaves is higher than in the region near the midrib. As a result, the midrib and mesophyll regions can be distinguished by chlorophylls distribution maps. When compared with healthy leaves, mildly and severely iron-deficient leaves had lower chlorophylls contents on the pseudo-color maps, making it more difficult to identify the positions of the midribs. Because of the difficulty of iron-ion transfer (Rustioni et al., 2018), the iron contents in venous regions of iron-deficient plant leaves are higher than in the inter-vein regions (Osório et al., 2014). This results in greater decreases in chlorophylls contents in the intervein regions because they are more susceptible to iron-deficiency stress than the midribs. In healthy leaves, the chlorophylls contents between veins are higher than those of the midrib and vein regions; iron deficiency causes lower chlorophylls contents between the veins. Consequently, chlorophylls distribution is uniform in Raman hyperspectral images, and veins cannot be identified.

The advantage of Raman spectroscopy is that it allows for single-spectrum, multi-component analysis. Simultaneously, a distribution map of the relative lipids content near the midrib was generated based on the characteristic lipids peak at 1444cm^−1^. Iron deficiency can cause a decrease in leaf lipids content, as shown in **Figure 3D**. There are two main reasons for this: iron stress reduces the soluble lipids content of the epidermis (Fernández et al., 2008); however, it also reduces the lipids content of the thylakoid membrane. Furthermore, the lipids content of the midrib was much lower than that of the mesophyll region. This could be because there are no chloroplasts in the midrib, resulting in reduced lipids contribution from thylakoid membranes. Consequently, in **Figure 3D**, there is a clear difference in lipids contents between the midrib and the mesophyll. This results in the pseudo-color map of lipids exhibiting relatively consistent venation distribution in the visible light image in **Figure 2B**.

### 3.3 Spatial distribution of metabolites in the leaf-vein region

**Figure 4:**
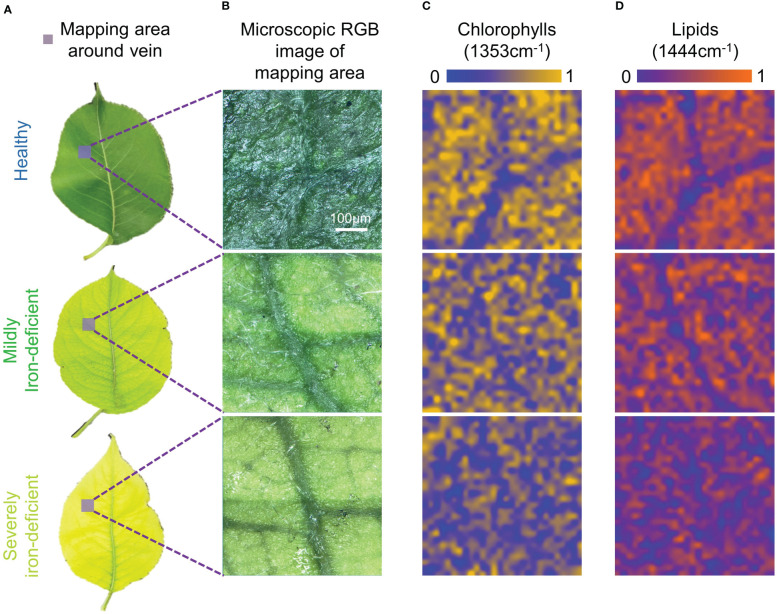
Visible light images and Raman spectral images of vein-region components of leaves with differing degrees of iron deficiency. **(A)** Visible light images of healthy and iron-deficient leaves; **(B)** Microscopic visible light images of the vein-mapping regions; **(C)** Pseudo-color maps of chlorophylls spatial distribution in the vein-mapping regions; **(D)** Pseudo-color maps of lipids spatial distribution in the vein-mapping regions.

**Figure 4** depicts the results of a similar pseudo-color map analysis of the area near the leaf veins. Like the midrib region, healthy leaves had higher chlorophylls contents in the mesophyll and lower contents in the veins. Because of differences in chlorophylls content distributions, it is possible to see similar structural textures of the leaf in **Figure 4C** as in **Figure 4B**, allowing a clear distinction between the veins and mesophyll regions. In **Figure 4C**, the chlorophylls content of the mesophyll gradually decreases with the degree of iron deficiency in mildly and severely iron-deficient leaves. The difference in chlorophylls contents between mesophyll and veins in leaves was reduced as the degree of iron deficiency increased, resulting in an unclear chlorophylls distribution profile in **Figure 4C**. Consequently, a leaf texture structure similar to that shown in **Figure 4B** is not visible. Similarly, **Figure 4D** depicts the lipids content distribution in the vein-mapping region. The lipids content distributions in the vein- mapping areas of the three types of leaves were more uniform than in the midrib-mapping area. This could be because the veins are smaller in size, resulting in a higher proportion of mesophyll.

### 3.4 Normalized intensities of chlorophylls and lipids within the mapping area

**Figure 5:**
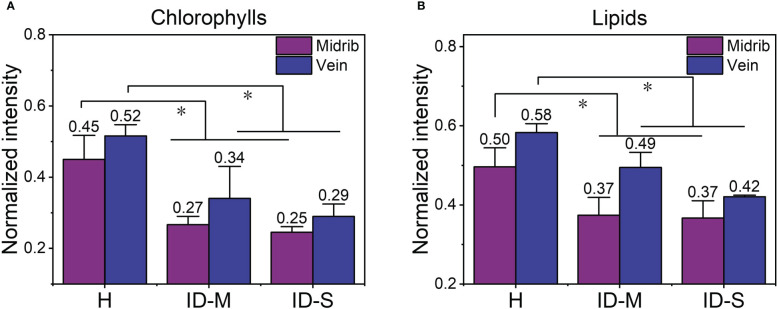
The average normalized intensity of chlorophylls and lipids in the mapping regions: **(A)** chlorophylls; **(B)** lipids. *, *p ≤ 0.05*. H, healthy leaves; ID-M, mildly iron-deficient leaves; ID-S, severely iron-deficient leaves.

The results of the preceding analyses show that micro-Raman maps can visualize the metabolites (detected chlorophylls and lipids) in leaves with varying degrees of iron deficiency. The average normalized intensities of chlorophylls and lipids in the mapping areas in **Figures 3**, **4** were calculated to more clearly quantify changes in the relative contents of chlorophylls and lipids with respect to the degree of iron deficiency in the mapping area; these results are shown in **Figure 5**. In iron-deficient leaves, including mildly and severely iron-deficient ones, the two substances in the midrib and vein decreased significantly compared with those in healthy leaves.

The mapping method was also found to be more stable than the single-point acquisition method. The RSD of spectral characteristic peak intensity of all single points in each leaf scanning area represents the inaccuracy of single-point acquisition method. At the same time, the RSD of the characteristic peak intensity of the average spectrum of the scanning areas of the three samples in each category (healthy, mildly iron-deficient, and severely iron-deficient) represents the inaccuracy of mapping method. As shown in the **Table 2**, the RSD of mapping is much smaller than that of single-point acquisition measurement, so mapping method can improve the accuracy and consistency of the results.

**Table 2:** The RSD calculated in the single-point and mapping acquisition methods.

	Method		Single-point		Mapping
	RSD	Sample 1	Sample 2	Sample 3	
	Category				
C h l o r o p h y l l s	Healthy	0.521133	0.265841	0.237239	0.061481
	Mildly	0.668272	0.428599	0.546086	0.263762
	iron-deficient				
	Severely iron-deficient	0.435153	0.383717	0.412524	0.120858
L i p i d s	Healthy	0.343743	0.416906	0.361109	0.038703
	Mildly				
	iron-deficient	0.356453	0.337559	0.343417	0.077702
	Severely				
	iron-deficient	0.334797	0.141416	0.500061	0.009228

## 4 Discussion

In this paper, the feasibility of using the micro-Raman spectral imaging method for detecting metabolites in iron-deficient leaves was preliminarily explored, and visual detection of changes in the distribution of leaf metabolites caused by iron deficiency was discussed. Although the Raman spectral characteristics of chlorophylls and lipids were not robust, we could still identify them and relate temporal and spatial variations of their relative contents with the degree of iron deficiency by Raman spectroscopy. We found that iron deficiency resulted in decreased chlorophylls and lipids contents in leaves, which was more pronounced in the mesophyll regions.

Iron deficiency in pear trees can result in significant yield reductions and even death. As a result, it is critical to determine the physiological state of iron deficiency in pear leaves accurately and precisely. Existing measurement methods primarily focus on measurement of total iron content in leaves and reflectance spectroscopy. The “chlorosis paradox” suggests, however, that total iron content in leaves cannot accurately reflect the physiological state of iron deficiency in plants, and the reflectance spectrum cannot specifically identify changes in leaf metabolites caused by iron deficiency. Compared with ICP-ES, the Raman spectral imaging method proposed in this paper can visualize the physiological state of iron deficiency in pear leaves with greater accuracy. It provides a method for visual characterization of specific substances for studying the mechanisms of plant responses to iron deficiency. Furthermore, the ability to recognize temporal and spatial variations in metabolite contents is expected to make different nutritional stresses distinguishable. Plant nutrients such as nitrogen, magnesium, and iron can cause leaf chlorosis, but there are subtle differences. The distribution of chlorophylls in leaves is expected to distinguish stress because of iron deficiency, magnesium deficiency, and nitrogen deficiency (Bertamini et al., 2002; Tremblay et al., 2012; Rustioni et al., 2018). These distinctions provide an opportunity for using Raman spectroscopy for nutrient stress discrimination. Future research will be focused on the use of Raman spectroscopy to diagnose specific nutrient stresses in plants.

Plants also contain many fluorescent chromophores. The weak Raman characteristics of many iron-deficiency-related substances may be obscured because of strong background interference by fluorescence in leaf Raman spectra. Fortunately, we can detect changes in chlorophylls and lipids contents in pear leaves caused by iron deficiency. However, in the study of iron-deficiency mechanisms in leaves, providing only temporal and spatial variation of these two metabolites is insufficient; detection of variation in trace substances is also required. Determining how to reduce the strong fluorescence background and investigate high-resolution and high-sensitivity Raman mapping technology for iron-deficiency-related metabolite content maps is a critical and difficult task. In future work, shifted excitation Raman difference spectroscopy (SERDS) can be used to remove fluorescence interference by changing the acquisition method (Theurer et al., 2021); a Fourier-transform Raman spectrometer excited by near-infrared light at 1064 nm or an ultraviolet micro-Raman spectrometer can also be employed to avoid fluorescence background interference (Gallimore et al., 2018; Nazari and Holtz, 2018). However, increasing the spectral resolution of the micro-Raman spectrometer can improve its ability to detect more substances in one measurement. In terms of operation time, existing micro-Raman spectroscopy relies on the point-scanning mapping mode, which takes a long time. More advanced Raman techniques could be used to solve this problem. The spectral imaging properties of coherent anti-Stokes Raman spectroscopy (CARS) and stimulated Raman spectroscopy are excellent (Hu et al., 2019; Xu et al., 2022). CARS is used to study anti-Stokes scattering, which not only reduces integration time but also significantly reduces the influence of fluorescence, improving mapping quality and speed. Similarly, because of the two-photon resonance effect, stimulated Raman spectroscopy increases the cross-section of Raman scattering and excitation efficiency, which can significantly improve the signal-to-noise ratio (SNR) and avoid fluorescence interference. High SNR means faster mapping speed, which is desirable when scanning larger areas. Using the techniques described above, it should be possible to detect more iron-deficiency-related metabolites in addition to chlorophylls and lipids and to fully utilize Raman single-spectrum, multi-component analysis.

## 5 Conclusion

Pear trees are grown widely and are valuable economic crops. Because the trees are iron-sensitive, iron deficiency is a common problem in pear cultivation, particularly in calcareous soils. Existing methods for determining iron deficiency in plants are destructive, necessitate complicated sample-preparation procedures, and do not accurately reflect the physiological state of plants suffering from iron deficiency. The Raman spectral imaging detection method proposed in this paper can detect iron deficiency on a microscopic scale without pre-processing and can accurately, non-destructively, and rapidly visualize changes in the relative content distributions of chlorophylls and lipids in pear leaves. To the best of our knowledge, this is the first use of Raman spectroscopy to investigate iron deficiency in pear trees. We have developed a new method of microscopic spectral image characterization for the study of physiological changes in pear leaves during iron deficiency. In the future, Raman spectroscopy could be used to study iron deficiency in other plant species. Additional characteristic peaks on the Raman spectrum for characterizing other substances will be mined with the advantage of single-spectrum, multi-component analysis. The metabolite-specific changes caused by iron deficiency in plants will then be studied in significant detail on a microscopic scale. It will be helpful to understand the mechanisms of plant responses to iron deficiency.

## Data availability statement

The raw data supporting the conclusions of this article will be made available by the authors, without undue reservation.

## Author contributions

ZG: Conceptualization; Methodology; Software; Formal analysis; Investigation; Data Curation; Writing - Original Draft; Visualization; Project administration; CZ: Supervision; DD: Writing - Review & Editing; Funding acquisition; SL: Resources; XW: Investigation; YG: Data Curation; LJ: Writing - Review & Editing; Funding acquisition. All authors contributed to the article and approved the submitted version.
